# From nets to networks: tools for deciphering phytoplankton metabolic interactions within communities and their global significance

**DOI:** 10.1098/rstb.2023.0172

**Published:** 2024-07-22

**Authors:** Charlotte Nef, Juan José Pierella Karlusich, Chris Bowler

**Affiliations:** ^1^ Institut de Biologie de l’École Normale Supérieure (IBENS), École Normale Supérieure, CNRS, INSERM, PSL Université Paris, Paris 75005, France; ^2^ Research Federation for the study of Global Ocean Systems Ecology and Evolution, FR2022/Tara Oceans, Paris 75016, France; ^3^ FAS Division of Science, Harvard University, Cambridge, MA 02138, USA

**Keywords:** phytoplankton, biotic interactions, symbioses, metagenomics, *Tara *oceans

## Abstract

Our oceans are populated with a wide diversity of planktonic organisms that form complex dynamic communities at the base of marine trophic networks. Within such communities are phytoplankton, unicellular photosynthetic taxa that provide an estimated half of global primary production and support biogeochemical cycles, along with other essential ecosystem services. One of the major challenges for microbial ecologists has been to try to make sense of this complexity. While phytoplankton distributions can be well explained by abiotic factors such as temperature and nutrient availability, there is increasing evidence that their ecological roles are tightly linked to their metabolic interactions with other plankton members through complex mechanisms (e.g. competition and symbiosis). Therefore, unravelling phytoplankton metabolic interactions is the key for inferring their dependency on, or antagonism with, other taxa and better integrating them into the context of carbon and nutrient fluxes in marine trophic networks. In this review, we attempt to summarize the current knowledge brought by ecophysiology, organismal imaging, *in silico* predictions and co-occurrence networks using 'omics data, highlighting successful combinations of approaches that may be helpful for future investigations of phytoplankton metabolic interactions within their complex communities.

This article is part of the theme issue ‘Connected interactions: enriching food web research by spatial and social interactions’.

## 1. Introduction

### (a) Metabolic interactions in the ocean microbiome

Oceanic microbial planktons, which we define as comprising viruses, archaea, bacteria, unicellular eukaryotes (protists, fungi and algae) and small metazoans, form a vital component of the planet as we know it. They dominate life in the world’s oceans in terms of biomass, organism abundance and diversity [1–3], compose the core of marine trophic networks [4,5] and have major impacts on multiple biogeochemical cycles. The critical roles of these organisms are mainly explained by the fact that a large fraction of them are photosynthetic primary producers. Photosynthetic plankton organisms, or phytoplankton, consist of unicellular organisms of diverse evolutionary history and ecology and they lock up almost as much atmospheric carbon (CO_2_) as plants do on land [6,7]. While it is now generally accepted that plankton interact within complex communities, it has long been thought that phytoplankton growth was constrained by a mix of ambient physical and chemical fluctuations that could favour select assemblages of species at specific locations and times of the year. Phytoplankton were as such considered fully autotrophic organisms, like higher plants [8]. This idea was largely fuelled by biases in algal culturing techniques, in which organic substances were present, for instance soil extracts [9]. Later on, laboratory and field experiments showed that the availability of exogenous compounds, in particular mineral nutrients, and their biological requirement by phytoplankton, were important regulators of oceanic primary production. This gave rise to appreciation of the C : N : P stoichiometry of phytoplankton; the Redfield ratio [10], coined after Alfred Redfield (Woods Hole Oceanographic Institution) who was considered one of the most fundamental parameters in plankton biogeochemistry. This concept is still used today to study and model phytoplankton nutritional responses, although the ratio turns out to be more variable than what was first estimated owing to biological variability in N : P proportions [11]. Starting in the 1950s, observations of phytoplankton blooms and temporal successions in the environment led researchers to posit that physical parameters and the availability of inorganic nutrients could not fully explain successions of very close species, but that other growth factors such as vitamins, which may be organic and provided by other members of the community, could be important as well [12]. In addition, the role of phytoplankton in the ecosystem is further complicated owing to the presence of mixotrophy (see Box 1), which expands the traditional view of purely phototrophic phytoplankton [13]. Mixotrophy covers: (i) phytoplankton taxa capable of phagotrophy of bacteria and small protists, (ii) phytoplankton species living in symbiosis with a heterotrophic host (photosymbioses), and (iii) ‘heterotrophic’ organisms that can temporarily retain functional chloroplasts from their ingested algal preys (kleptoplastidy). The efficient recycling of organic and inorganic nutrients in these trophic modes can provide a critical advantage under oligotrophic conditions.

Box 1.
**Glossary.**

**Amensalism:** Long-term association between two organisms, which is detrimental to one and neutral to the other.

**Autotrophy:** Mode of nutrition through which an organism is able to produce complex organic compounds to obtain energy.

**Auxotrophy:** Inability of an organism to synthesize a compound required for its growth.

**Competition:** Interaction between two organisms that decreases the fitness of one by the presence of the other.

**Commensalism:** Long-term association between two organisms, which is beneficial to one and neutral to the other.

**Diazotrophy:** Capacity of some prokaryotes to fix atmospheric dinotrogen (N_2_) into a form more biologically available.

**Ectocrines:** Term relating to substances excreted in the environment by an organism that can influence the development of close neighbours and that can play major roles regarding plankton ecosystem dynamics.

**Heterotrophy:** Mode of nutrition through which an organism must take up exogenous compounds to obtain energy.

**Intergenic regions:** DNA sequence located between genes.

**Introns:** Sequence within a gene that is not expressed or involved in the formation of the final RNA.

**MAGs:** Metagenome-assembled genomes. Genomes reconstructed from metagenomic reads using specific algorithms.

**Metabarcoding:** Molecular technique for identifying species by amplifying and sequencing fragments of conserved universal genes (typically from the nuclear small subunit rDNA) and comparing them with known references at the level of bulk communities.

**Metabolic interaction:** Interaction between organisms at the level of the compounds they produce. As an example, one metabolite produced by one organism can be taken up by another and turned into biomass, or one organism can excrete a compound that is toxic to the other.

**Metabolites:** Intermediate or end-products of cellular metabolism.

**Metabolomics:** Process by which all the metabolites of an organism or biological sample are identified and quantified.

**Metagenomics:** Process of getting information from the genes of organisms within a community.

**Metatranscriptomics:** Process of getting information from the gene expression levels (typically mRNA) of organisms within a community.

**miTAGs:** Metagenomic illumina tags. Gene assemblies derived from metagenomes sequenced with an Illumina platform and taxonomically assigned by comparing with reference databases. These sequences theoretically allow us to tease apart the effect of PCR bias versus copy number in the patterns of V9-18S metabarcoding.

**Mixotrophy:** Mode of nutrition combining autotrophy and heterotrophy for carbon/energy acquisition.

**Mutualism:** Long-term association between two organisms that is beneficial to both partners.

**‘Omics:** Wide variety of methods aiming at characterizing and quantifying the pools of biological molecules within organisms (e.g. genomics, transcriptomics and metabolomics).

**OTU:** Operational Taxonomic Unit, a pragmatic definition of a collection of closely related organisms.

**Parasitism:** Long-term association between two organisms, which is beneficial to one and detrimental to the other.

**Phycosphere:** Space surrounding algal and cyanobacterial cells that is highly enriched in metabolic compounds (either by passive diffusion or active mechanisms) originating from metabolic activity.

***psbO*:** Gene encoding the manganese-stabilizing polypeptide of the photosystem II oxygen-evolving complex, an essential link of the photosynthesis pathway found in all photosynthetic organisms (eukaryotes and prokaryotes) and apparently present universally in one or two copies per genome.

**Rhizosphere:** Zone surrounding the root of vascular plants that is enriched in secreted molecules.

**SAGs:** Single amplified genomes. Genomes obtained by isolating individual cells, massively amplifying and sequencing their DNA.

**Symbiosis:** Any type of close and long-lasting association between two organisms.

**Syntrophy/cross-feeding:** Phenomenon of one organism feeding on the metabolic product(s) of another one.

Furthermore, many microbial plankton are in fact unable to synthesize some of the compounds necessary for their growth, in which case they are termed ‘auxotrophic’ for the metabolites concerned. Very often, the missing compound is acquired by exploiting the resources produced by other organisms in the community, or ‘ectocrines’ (see Box 1). This non-predatory, mutualistic association among community members is designated as cross-feeding or syntrophy. More generally, we observe what we call ‘labour division’ between microbial members of complex communities, where the different partners are in close metabolic interaction through the exchange of one or more metabolic compounds, in a relationship that is often mutually advantageous for all partners. In this way, the Black Queen Hypothesis [14] emerged as a concept explaining evolutionary forces driving genome sizes in microbes. It is based on the observation that some important metabolic functions are not encoded by organisms that need them, while they display large population sizes. Hence, there must be a selective advantage outweighing the cost associated with the loss of function. In the context of complex communities, such as what we observe for marine plankton, some 'helper' individuals that do not necessarily account for large proportions of the population remain able to provide the important metabolic function to the majority, the ‘beneficiaries’. The said metabolic function must however be easily accessible to the community members, or ‘leaky’. In other terms, the beneficiaries have a selective advantage, induced by a reduction in genome size and optimization of metabolic efficiency, as long as there are enough helpers in the community. As such, there is a functional dependency of community members on helpers that must themselves derive some degree of benefit for providing the function themselves.

Interactions between species may occur through predation, pathogenesis or symbiosis (defined as any type of close and long-lasting association—either positive or negative—between organisms, such as parasitism, commensalism or mutualism [15]). While several characteristics can be used to describe biotic interactions such as these (e.g. specialization level, strength or type), many of them are not yet fully understood mechanistically. Recent studies using global genomic approaches to characterize plankton distribution patterns have demonstrated that these communities remain tightly intertwined by their interactions and metabolic relationships [16,17]. The effect of metabolic interactions may even leave a track on the genome of the organisms, exemplifying their evolutionary importance, such as the endosymbiotic events and gene transfers that led to the appearance of mitochondria and chloroplasts in eukaryotic cells and ultimately to phytoplankton diversity as we know it today. As such, some metabolic interactions can be considered as a kind of symbiotic relationship.

Though shown for simple communities [18,19], demonstrations of these concepts of metabolic interaction on larger community or ecosystem scales are more limited [20–22]. This is notably owing to the difficulties of following metabolite uptake/exchange as it is not possible to connect a precise metabolite with its producer(s) and consumer(s) within complex communities and because organisms can secrete a vast number of compounds [23,24], with some of them in extremely low quantities. Appropriate protocols must therefore be followed for sample transportation and preparation [25]. One also needs to keep in mind that characterizing these exchanges requires compromise between resolution and coverage, as their nature is dynamic and needs to be integrated across space and time. This review aims to focus on phytoplankton interactions with their associated plankton partners—especially prokaryotes—and on how these interactions enable metabolic innovations to emerge. We will describe the approaches that have contributed to some of the major advances leading to their characterization, with a particular focus on the insights brought by the extremely deep sequencing data from *Tara* Oceans. These include reductionist approaches—mostly at the species level—and more recent *in silico* methods to study at the (small) community level, as well as exciting new approaches combining sets of complementary analyses that broaden the perspectives of the field.

## 2. Reductionist approaches to decipher the dynamic cues for specific interactions

### (a) Culturing-based approaches to narrow down interactions

When we zoom in, many metabolic exchanges take place in the ‘phycosphere’ (see Box 1) [26,27]. This particular microhabitat is characterized by altered oxygen and pH levels compared with the ambient medium, and it is a place where the compounds secreted by phytoplankton cells form a chemical gradient—that extends several cell diameters from the cell surface—that can favour some prokaryotic communities and be detrimental to others [26,28]. All these particularities make the phycosphere the aquatic analogue of the rhizosphere of terrestrial plants. When trying to characterize metabolic interactions between phytoplankton and a plankton partner, traditional methods involve either one or both of co-culturing the organisms and comparing their growth patterns with separate cultures; and cross-culturing, consisting of adding the culture filtrate of one organism to the culture medium of the other. From there, a diverse set of physiological and chemical measurements can be undertaken (e.g. growth rate, photosynthetic capacity, elemental and metabolite composition).

The biochemical bases of phytoplankton–bacteria interactions among natural communities have been investigated in many studies, mostly involving the predominant phytoplankton groups in the oceans. Such dynamics of interactions have been investigated in natural isolates of the diatom *Pseudo-nitzschia multiseries* [29]. Co-cultivating the diatom with individual bacterial strains found in the isolates and synthetic seawater lacking organic carbon exhibited contrasting results. While some strains were found to be lethal to the diatom, some—including *Sulfitobacter* strains—promoted its growth but not that of other diatom species, highlighting the remarkable specificity of phytoplankton–bacteria interactions. Transcriptome analyses and measurements in the surrounding environments were conducted on both species grown either in isolation or in co-culture and indicated that when grown together *Sulfitobacter* increases nitrate uptake and ammonium secretion, which is preferentially used by the diatom rather than nitrate. Additionally, analyses of environmental data showed that the expression level of genes involved in nitrogen metabolism in prokaryotes correlated with those of diatom ammonium transporters [30], suggesting a potential uptake of prokaryotic NH_4_^+^⁠ by diatoms. Most interestingly, the co-culture seemed to induce the excretion of the plant hormone indole-3-acetic acid (IAA) and its precursors, which was confirmed to enhance *P. multiseries* growth by *Sulfitobacter*. Moving forward from the lab to the environment, the analysis of different sampling stations revealed that IAA biosynthetic pathways were transcribed in natural *Roseobacter* populations, which was corroborated by results from targeted metabolite analyses showing the presence of extracellular IAA in surface seawater and deep chlorophyll maxima of five North Pacific Ocean stations where high phytoplankton abundances were detected. Such results highlighted the specificity of metabolites in mediating phytoplankton–bacteria interactions, and particularly, that some level of recognition of the molecules may exist, allowing the identification and exclusion of non-beneficial organisms and underpinning a long evolutionary history of association between the partners.

In some cases, a mutualistic exchange of metabolites can take place until a certain breaking point is reached, where the interactions shift to being detrimental. One such example explored the dynamics of interactions between the widespread haptophyte microalga *Emiliania huxleyi* and bacteria from the roseobacter clade (Alphaproteobacteria), which are usually found together during blooming events of the microalga [31]. The authors used bioinformatic and chemical searches to identifying potential metabolites involved in the interaction. Cross-culturing different bacteria strains with various concentrations of *p*-coumaric acid, a product of lignin degradation from plant cell walls, revealed specific stimulated production of algicidal compounds for the alga and potentially originating from *p*-coumaric acid metabolism by *Phaeobacter gallaeciensis*. Two distinct phases for the interaction were therefore deduced, with a first mutualistic interaction between healthy algal cells and the bacteria, where the latter can promote algal growth [32]. Once a threshold of *p*-coumaric acid is reached, probably resulting from algal population peaks or senescence during blooming events, the synthesis of algicidal compounds from the bacteria is induced, shifting the relationship from mutualism to pathogenesis.

The interaction between nitrogen-fixing symbionts and their eukaryotic hosts is a good model to study metabolic interactions owing to their exchange of nitrogen and carbon compounds, such as the association between the diatom *Hemiaulus hauckii* (host) and the nitrogen-fixing cyanobacterium *Richelia euintracellularis* (endosymbiont) in which the cyanobacterium is uncultivable. The heterologous expression of genes in model laboratory organisms was crucial to identify multiple protein functions from the endosymbiont that are involved in organic carbon import from the host, explaining how the symbiont is able to sustain high rates of dinitrogen fixation [33]. The importance of these proteins was also confirmed by the detection of elevated transcript abundances in natural populations.

Another way to characterize potential metabolic interactions between plankton partners resides in the use of artificial barriers to physically separate the organisms, which is preferable for following the fluxes of compounds and their identification as well and also better controls the conditions in each microenvironment [34]. While culturing-based approaches represent the definitive proof of an interaction, caution is warranted when considering the (co-)culture conditions [35]. Indeed, these may have a significant impact on the partner metabolisms as they may be highly artificial (e.g. with nutrient levels far exceeding the concentrations found in the open ocean). These aspects must therefore be taken into account when drawing conclusions and extrapolations must therefore be drawn by taking these aspects into account.

### (b) Benefits of labelling and imaging

One step beyond the characterization of dynamics and cues is the labelling of compounds in order to identify and better quantify the fluxes of matter and their assimilation within cells and cellular components. The nitrogen-fixing cyanobacterium *Trichodesmium* forms large colonies associated with a multitude of bacteria, and stable isotope incubations of field-sampled *Trichodesmium* colonies indicated that new nitrogen is preserved in the colonies (e.g. dinitrogen fixation and recycling dominate, whereas nitrogen loss via denitrification was negligible) [36]. Model calculations of nitrogen gradients based on these incubations suggest complete nitrate depletion in the centre of the colonies but up to sixfold higher ammonium concentrations at their centre compared with the surrounding seawater. One major challenge is to distinguish the labelling fingerprints of different populations, which can be achieved by combining them with specific peptides or metagenomics [37,38] (see Box 1).

Characterizing the fluxes of compounds between interacting partners may require visual investigation of their spatial distribution using specific techniques. For instance, fluorescence *in situ* hybridization (FISH)-based methods using different probes enabled the detection of distinct bacterial taxa associated with a diatom and a dinoflagellate partner [39] (table 1). Such an approach allowing the identification of organisms can be extremely powerful, especially when combined with secondary ion mass spectrometry (SIMS) and stable isotope probing at the nanometre-scale (nanoSIP) to permit elevated spatial resolution (typically around 50 nm), high sensitivity and specificity [40] (table 1). One example is the exploration of a symbiotic relationship between the nitrogen-fixing cyanobacterium UCYN-A and a haptophyte alga. These symbioses are characterized by genome reduction and coevolution between the cyanobacterium and its eukaryotic host. Indeed, UCYN-A has a small genome and such extreme metabolic streamlining that it has lost the ability to fix CO_2_ or perform oxygenic photosynthesis, as do typical cyanobacteria [45]. This symbiosis was studied by screening North Pacific Ocean seawater samples coupled with quantitative PCR assays for the nitrogenase (*nifH*) gene specific to UCYN-A [42], and visualization and quantification of the cell-specific uptake of select isotopes by using a halogenated *in situ* hybridization nanometer-scale SIMS (HISH-SIMS) (table 1). Results confirmed the active transfer of fixed nitrogen to the partner cell associated with UCYN-A. This association was further explored and visualized using catalysed reporter deposition fluorescence *in situ* hybridization (CARD-FISH) coupled with nanoscale secondary ion mass spectrometry (nanoSIMS) and isotope probing at the single-cell level (SC-SIP) to disentangle the exchange of metabolites of individual associations when supplied with external sources of dissolved inorganic nitrogen [41] (table 1). While the host did not assimilate the inorganic nitrogen, it responded with enhanced carbon and N_2_ fixation. In the same vein, symbioses between diatoms and nitrogen-fixing cyanobacteria of the genus *Richelia* have been recently studied by isotope labelling coupled to large-geometry-SIMS (LG-SIMS) and nanoSIMS imaging to study N_2_ and C fixation activity of single cells within different populations [43] (table 1). Such a method was previously applied to explore nutrient dynamics in the mutualistic partnership between a vitamin B_12_-dependent mutant of the green alga *Chlamydomonas reinhardtii* and the B_12_-producing heterotrophic bacterium *Mesorhizobium japonicum* [46].

**Table 1.** Principle and example application of methods used for labelling and imaging.

method	principle	reference
SIMS	secondary ion mass spectrometry; a technique consisting of analysing the composition of a sample by bombarding it with ions to produce secondary ions that will be analysed.	[40]
nanoSIMS	nanoscale secondary ion mass spectrometry; it allows to get information about the isotopic and elemental composition of a sample, with a nanoscale resolution down to ~50 nm. It can be applied for instance to image metabolically active cells.	[41]
HISH-SIMS	halogen *in situ* hybridization nanometer-scale secondary ion mass spectrometry; it combines isotope probing with imaging in a sensitive way. It can help determine and quantify the individual contributions of active microorganisms.	[42]
LG-SIMS	large-geometry secondary ion mass spectrometry; it follows the same principle as nanoSIMS with a higher sensitivity and precision of isotope ratios.	[43]
nanoSIP	nanometer-scale stable isotope probing; the combination of nanoSIMS with stable isotope labelling; it provides a distinctive means to quantify net assimilation rates and stoichiometry of individual cells in both low- and high-complexity environments.	[40]
SC-SIP	single-cell stable isotope probing; it uses nanoSIMS or Raman spectroscopy to perform the tracking of isotope tracers within cells and cellular components in a spatially-resolved manner.	[40]
FISH	fluorescence *in situ* hybridization; a molecular technique using fluorescent probes that bind to specific parts of a nucleic acid sequence with a high degree of sequence complementarity. It can be used to detect, identify and count microorganisms without requiring culture.	[39]
CARD-FISH	catalysed reporter deposition fluorescence *in situ* hybridization; improvement of traditional FISH with higher signal sensitivity by using horseradish peroxidase labelled probes and fluorescently labelled tyramide that is converted into a radical intermediate.	[41]
smFISH	single-molecule messenger RNA *in situ* hybridization; it follows the same principle as FISH but targeting mRNA or other long RNA.	[44]

These methods can also be embedded within high-throughput systems to study natural communities. One cutting-edge example is the application of single-molecule messenger RNA *in situ* hybridization (smFISH), where the signal intensity is proportional to the gene expression level, to quantify the proportion of active viruses within cells of *E. huxleyi* during a bloom event, targeting both the host and virus transcripts at the subcellular level [44]. By tracking the infection dynamics using high-throughput imaging flow cytometry, the authors showed that in natural assemblages viral infection reached only 25% of the population despite synchronized bloom demise, pointing out the coexistence of infected and non-infected subpopulations (table 1).

Regarding cross-feeding, however, where a labelled metabolite is transferred from the primary producers to other members of the community, the application of such analyses may be ambiguous. Musat *et al*. proposed several aspects that should be considered to make the most out of nanoSIP, such as the shortest possible incubations, the quantification of the relative level of incorporation of the label and the use of genomic data to constrain metabolic potential [47]. As the costs decline, we expect that the use of SC-SIP in the field of microbial ecology will expand, providing a better understanding of the molecular bases of (phyto)plankton metabolic interactions [40].

### (c) General limitations for community-level studies

**Figure 1.**
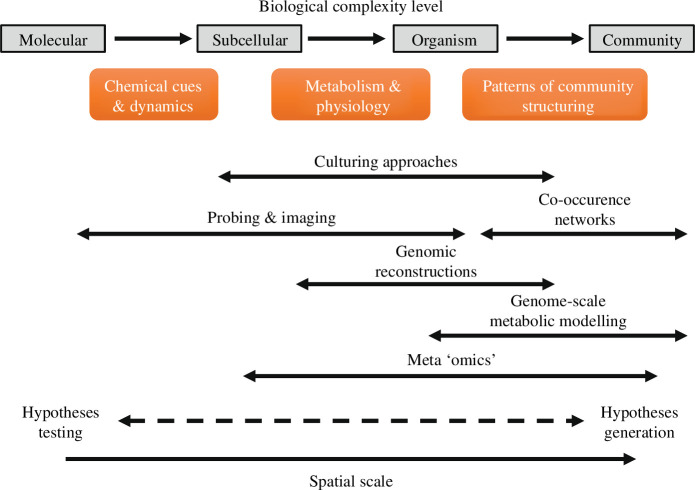
Overview of the diverse range of approaches to disentangle phytoplankton metabolic interactions, their corresponding biological and spatial resolutions and their ecological meaning. Culturing approaches, probing and imaging are best suited for exploring interactions at small spatial scales or for very simplified associations and for testing hypotheses, whereas genomic reconstructions and meta-omics are useful for investigating larger patterns of organization and biological signals within small communities. Finally, co-occurrence networks and genome-scale metabolic modelling are valuable tools to identify large-scale patterns of association within communities and to generate new research questions to be explored experimentally.

Although reductionist approaches appear necessary to generate testable hypotheses regarding the biological and chemical mechanisms at play and are particularly needed to formally demonstrate that a specific element is exchanged, one major limitation is that they are not well suited for informing about interactions and emergent properties—properties resulting from the behaviour of a system that is ‘larger than the sum of its parts’—at the level of communities. Moreover, the vast majority (~99%) of microorganisms remain uncultured [48], and laboratory-based studies can fail to grasp the environmental context in which natural interactions occur because many microbial metabolic processes occur in the context of communities and not simple cultures. Overall, to fully understand community processes, there has to be a balance between a focused approach to divide complex processes into smaller ones that can be better observed, quantified and characterized mechanistically, and holistic approaches focusing on the interconnections between system components (figure 1). These holistic approaches to studying natural habitats more directly can yield complementary data to help deduce the interactions.

## 3. *In silico* approaches for community-level studies

### (a) Plankton sampling expeditions in the twenty-first century

As stated previously, collecting information from laboratory-based experiments remains biased by the fact that we are able to grow only a small fraction of the organisms present in a natural sample. Significant advances in the field have been made with the development of ‘omics techniques, which have revolutionized our understanding of microbial communities by disentangling complex information encrypted in a single ecosystem, circumventing the need to grow natural samples in the laboratory. Typical ‘omics data in phytoplankton community ecology encompass metabarcoding, metagenomics, metatranscriptomics and metabolomics (see Glossary). While the first technique enables the simultaneous identification of different species in a given environment and provides estimates of species diversity, the others give insights about the functional characterization of organisms (i.e. their biological role) by providing clues about how they respond and adapt to changes in their environment and are therefore complementary [49]. A few global projects have tried to compile a holistic view of microbial plankton ecosystems by establishing an inventory of their diversity and abundance (we invite the interested reader to see a detailed review of the history of phytoplankton exploration in [50]). Among them, three major projects brought to light the extreme diversity of plankton organisms through the exploration of their genomic information.

The first was the Global Ocean Sampling Expedition (GOS; 2004–2008) performed on the sailing vessel *Sorcerer II* and led by J. Craig Venter, which collected genomic information from marine bacteria, archaea and viruses using environmental DNA sequencing. Sampling was undertaken from the northwest Atlantic Ocean to the eastern tropical Pacific Ocean and yielded more than 6 million protein-coding genes, dramatically expanding the information that was available in public databases [51,52]. Novel techniques to cluster full-length protein sequences from fragmented sequences were developed, enabling improved methods to group the related sequences and predict their putative functions. The project provided insights into the distribution and expression levels of phage and cyanobacterial photosynthesis genes in the ocean [53], and described for the first time the contribution of aerobic anoxygenic photosynthetic bacteria to plankton assemblages and the variations of their abundance and composition between different oceanic regions [54]. Most importantly, GOS represented the first global sampling effort investigating microbial and viral sequences in marine environments. The exploration of microbial metagenomic sequences during the first phase of the project identified more than 150 000 viral peptide sequences, representing around 3% of the total predicted proteins within the GOS dataset [55]. Furthermore, analysing the translated viral sequences allowed us to identify enrichment in host-specific functions such as photosynthesis and carbon metabolism.

The Malaspina Circumnavigation (2010–2011), led by Carlos Duarte, came later as a larger sampling project aiming to assess the state of the oceans and to specifically investigate life in the deep ocean using the oceanographic research vessel *Hespérides*. The research vessel sampled the tropical and subtropical Atlantic, Indian and Pacific Oceans, including their respective gyres, down to about 4000 m depth, over a period of 8 months. Among the main results are the first estimates of both the abundance and biomass of heterotrophic protists within the mesopelagic (200–1000 m) and bathypelagic (1000–4000 m) layers [56], showing a global biomass decrease with depth, and several sites—notably in the South Pacific Ocean—displaying exceptionally higher abundances than others in the bathypelagic zone. On the other hand, the project provided a first approximation of the load and nature of microplastic debris in the surface ocean [57]. One in every 10 samples was kept within the Malaspina Collection to be analysed by future scientists in anticipation of the development of new technologies in the next few decades [58].

The *Tara* Oceans project, launched in 2009, aimed at exploring global marine microbial ecosystems from the gene to the community level. The project developed a dedicated and highly standardized sampling protocol to investigate the photic and mesopelagic zones, by combining traditional (e.g. plankton net tows) and original methods (e.g. new methods to concentrate viruses). One major originality of the project was to bring together experts from a wide range of disciplines (e.g. molecular and systems biology, microbial ecology, genomics, informatics, biological and physical oceanography, modelling and data management). Different nucleic acid extraction processes were applied to obtain DNA and RNA from the different size fractions collected during the expedition (see [59] for a detailed description). These generated metagenomic and metatranscriptomic information, together with metabarcoding from amplicons of 16S and 18S rDNA, and sequencing was performed using Illumina platforms at Genoscope in Evry, France. The sequencing effort to perform 18S rRNA metabarcoding of the V9 region was considerable in order to overcome some of the limitations of metabarcoding-based approaches [59,60]. The 16S rDNA fragments derived from metagenomes were identified and assembled for generating 16S rDNA miTags (i.e. metagenomic Illumina tags, see Box 1) [61,62]. As with Malaspina, all processed data were made available to the scientific community. Overall, an unprecedented amount of around 50 million prokaryotic and 100 million microbial eukaryote genes have been surveyed by the *Tara* Oceans circumnavigations. An estimated 150 000 microeukaryote taxa were identified, of which only 10% were previously documented, and almost half of the expressed eukaryotic sequences did not display any similarity with known proteins and a non-negligible proportion resolved into newly described gene families [60]. Together with new gene and genome catalogues, these results make *Tara* Oceans one of the most successful global multidisciplinary explorations of plankton diversity so far [63]. All these projects highlighted the importance of plankton as being considered as complex and dynamic communities of organisms. As will be described in the following sections, the resources generated from the above three projects, together with others, have permitted a range of studies to explore interactions within plankton communities.

### (b) Generating ‘omics data for large-scale studies

A notable example is the global estimation of the *Symbiodinium* dinoflagellate, an endosymbiont of reef-building corals, using the V9 rDNA metabarcoding datasets from *Tara* Oceans expeditions, which suggested for the first time the existence of both free-living and host-associated populations outside the reefs [64]. Mapping a diverse range of reference transcriptomes from all *Symbiodinium* clades on the *Tara* Oceans global atlas of eukaryotic genes showed significant differences, particularly for nitrogen uptake and recycling between the predicted free-living and symbiotic populations, while another study identified the calcifying ciliate *Tiarina* sp. as a new host for a pelagic photosymbiosis [65]. Another analysis using V9 rDNA data revealed that photohosts were not only among the most abundant functional groups in the ocean, in particular radiolarians, but also dinoflagellates and members of Foraminifera and Ciliophora. Radiolarians have often been overlooked in traditional morphological surveys of plankton net-collected material because of their delicate gelatinous and/or easily dissolved structures, but microscope-based and *in situ* imaging studies have more recently shown that they are highly abundant [66,67]. Another example using ‘omics in phytoplankton metabolic interactions is the analysis of metagenomes and metatranscriptomes from *Trichodesmium* microbiomes that revealed specific functions of the epibiont community and synchronization of the cells potentially mediated by signalling molecules [68–70]. These studies open new perspectives regarding the integration of large-scale data to explore plankton interactions and their biological significance in marine ecosystems.

However, despite the vast amount of ‘omics resources that have been made available to the community these past decades, some biases and limitations remain. For example, most complete phytoplankton genomes and transcriptomes are from species isolated from North American and European oceanic regions and many are from coastal and freshwater environments, at the expense of open-ocean algae, which may be less easily accessible. Also, species that can be studied in the laboratory are not necessarily representative of their compatriots in the real world, as reflected in the first sequenced red alga *Cyanidioschyzon merolae*, an extremophile isolated from acidic hot sulphur springs and very different from most marine red algal relatives [71,72]. In addition, reference strains for genomic studies may have been cultured for several decades under nutrient-rich conditions in the laboratory, so the somatic mutations that occurred naturally during their time in culture may impair our understanding of their physiology [73,74]. On the other hand, many lineages comprising important genomic references (e.g. phaeophytes) represent minor components of marine plankton communities in the open ocean [1,3]. Studies involving metatranscriptomes containing sequences derived from non-model organisms represent bioinformatic challenges. The production of chimeric sequences and the inability to distinguish their taxonomic origins are recurrent difficulties in *de novo* assembly analyses. New methods are being developed that try to better distinguish the sequences corresponding to each partner of an interaction and to decrease the potential number of chimeras [75]. Finally, while the study of single marker genes (e.g. 18S rDNA) or functional genes can improve knowledge on the diversity and functions of lineages with well annotated cultured representatives, they necessarily provide fewer insights into taxa for which reference libraries from cultured species do not exist. This is, for instance, the case for dinoflagellates, which remain poorly represented in databases, notably owing to their elevated genome size and gene copy numbers (e.g. *Apocalathium malmogiense* can harbour up to 33 000 copies per cell of the 18S rRNA) [76], which largely overestimate their abundances using metabarcoding approaches.

### (c) Co-occurrence networks provide testable hypotheses

It has been observed that over large spatial scales the occurrence of organisms follows non-random patterns that result from a wide range of processes (e.g. habitat filtering, competition to access limiting elements, biotic interactions and neutral processes) [77]. These patterns can be disentangled to identify potential associations between organisms. To examine the frequency of species associations, co-occurrence networks derive from analytical methods typically based on meta-omics data, using specific sequences as proxies for organism abundances and identification of their taxonomy. These association networks are thus useful methods to capture emergent properties of the systems resulting from putative interactions and, by allowing the creation of assumptions about community assembly rules, they can provide testable hypotheses to explore biotic interactions and ecological niches [78]. In this way, the first global-scale plankton network of associations was generated using co-occurrence methods, based on 313 plankton samples from the *Tara* Oceans expedition derived from seven size fractions, covering a total of 68 sampling locations at two depths, and across eight oceanic provinces [16]. Using random-forest-based machine learning to tease apart the effect of abiotic and biotic factors revealed that 95% of the organism (OTU level, see Box 1) abundances were better explained by biotic factors. Network inference generated using Spearman and Kullback–Leibler dissimilarity measures allowed the prediction of 81 590 biotic interactions, with an overrepresentation of positive associations involving syndiniales and other previously undescribed parasites, and negative associations involving arthropods, providing new hypotheses to be tested in the laboratory to disentangle plankton interactions. A recent update of this work specifically focused on modelling the effect of predicted climate change on the global structure of plankton communities [17] by applying integrated niche modelling, i.e. inferring conditional dependencies between taxa and environmental parameters to infer a network, while computing the environmental optimum and niche tolerance of each OTU. Simulating the effect of environmental change was done by designing a network attack procedure, which consisted in progressively removing network nodes starting with the ones with the smallest environmental tolerance range, and computing the network robustness at each step to evaluate its vulnerability. This revealed biome-specific community responses to environmental change and predicted, within each community biome, the lineages most vulnerable to these changes based on a projected climate change scenario [79]. This study therefore highlighted specific groups as potential bioindicators for monitoring ocean change, which could be considered in future more targeted studies involving co-culture experiments and hypothesis testing. Interpreting correlation networks remains a difficult task that can introduce spurious correlations that deviate widely from far from reflecting the true biological signal, especially since stronger associations (e.g. competition or mutualism) are more easily demonstrated, and the lack of *a priori* knowledge about the mechanisms can bias results [80]. Distinguishing direct interactions from response to the same parameter/taxon can be done by including environmental factors as additional nodes and computing their associations with microbial taxa [81,82]. Overall, these methods need to be confronted with empirical data before being considered as being ground-truthed, but they represent valuable approaches to process large ‘omics datasets and drive hypotheses generation.

### (d) Uncovering interactions within communities using genomic reconstructions and metabolic modelling

The decrease in sequencing costs combined with the improvement of bioinformatic methods allowing the reconstruction of high-quality genomes from environmental samples have dramatically expanded the number of plankton genomes available and have provided insights into the genome sequences of species that are not yet cultivable. Currently, two major types of genomic data can be generated from natural samples: single amplified genomes (SAGs)—obtained by isolating individual cells, amplifying their DNA and sequencing their genomes, and metagenome-assembled genomes (MAGs)—generated by assembling metagenomic reads into larger fragments using specific algorithms and sequence signatures (see Box 1). The first MAGs were reconstructed from prokaryotic communities in an acid mine drainage site [83] using methods that have since been widely applied to hundreds of thousands of prokaryote populations, including from marine systems [84–88]. However these reconstructions are more difficult for eukaryotic phytoplankton—and eukaryotes more broadly—owing to their elevated genome size and complexity (e.g. introns, intergenic regions and repetitive elements) that make them problematic to assemble from environmental sequencing datasets, especially in the absence of a closely related cultured reference [89,90]. The identification and assembly of MAGs and SAGs raise new challenges pertaining to assembly and quality, concerning, for example, the minimum information acceptable for deposition in public repositories [89] and for standardized meta-data (e.g. environmental parameters) to improve their direct comparability to one another [91]. Nonetheless, new methods are being deployed that enable the reconstruction of MAGs, with the help of specific bioinformatic pipelines using marker gene phylogenies, GC content, tetra-nucleotide frequencies, depth of DNA sequence coverage and abundance across the samples [3,92]. In this way, recent databases based on the exploitation of *Tara* Oceans environmental samples and including the undersampled Arctic Ocean have been made available, including almost 2000 prokaryotic genomes [88], and among the >700 eukaryotic genomes reconstructed more than 270 genomes can be assigned to phytoplankton [93], unveiling exciting new research avenues.

However, functional annotation and characterization of such datasets are necessary steps to improve our mechanistic understanding of their ecological role within communities. While the integration of genomic information into co-occurrence networks appears to be particularly efficient for exploring the functional self-organization of microbial communities [94], genome-scale metabolic models represent a step further. By integrating a diverse set of ‘omics data and taking into account an organism’s metabolic network by incorporating biochemical reactions that can be associated to their respective genes and encoded enzymes, together with the application of formal mathematical formulations, they can be used to predict the state of the network in different growth conditions [95–97]. Reconstructing metabolic networks from environmental genomes would require expert knowledge on species that are often uncharacterized. Therefore, semi-automated approaches using reference metabolic reactions from pan-genomic collections to infer phenotypes represent a better alternative. As a solution to this problem, a first meta-metabolic model for unicellular phototrophic eukaryotes, named PhotoEukStein, has been recently proposed, that allows a fast and automated top-down derivation of genome-scale metabolic models (GSMs)—a network-based technique collecting all known metabolic information of a biological system (e.g. genes, reactions, enzymes and metabolites) and providing quantitative predictions for growth or fitness—directly from genomes [98]. The originality of the model lies in the combination of 16 metabolic models for marine photosynthetic protists covering the majority of their described taxonomic diversity. It allows a fast and automated top-down derivation of species-specific GSMs using the CarveMe algorithm [99], which can deal with partial genomes (e.g. MAGs). This algorithm was applied to a collection of 549 environmental genomes and transcriptomes, including 259 Oceans MAGs from marine phytoplankton and revealed both a taxonomic imprinting of the repertoires of metabolic reactions across the phytoplankton studied and the convergence of metabolic phenotypes of distantly related species within a given environmental context. While these methods remain exploratory, future development of such approaches and their application to environmental genomes will be pivotal to characterize phytoplankton functional traits and their biological role within communities, which will, in turn, sustain ecological models.

Recent studies have taken this approach a step further by inferring a global ocean association network based on genome activities that were inferred by integrating genome-wide abundance and transcript levels. As an example, the integration of *Tara* Oceans metagenomes and metatranscriptomes data from 71 samples allowed prediction of genome-scale interactions within prokaryotic communities [100]. The mapping of *Tara* Oceans on a database of 7658 genomes—including MAGs, SAGs and whole-genome sequences—was used as input to perform a species metabolic coupling analysis to infer the level of competition and metabolic interdependencies between species within co-active consortia. The significant advantage of this approach is that it circumvents the need for prior knowledge on the metabolic characteristics of species or optimality assumptions, and solely requires species identification and genomic sequences to map all possible interspecies metabolic exchanges and provide a likely, biologically meaningful prediction of metabolic interdependencies [101]. In addition to supporting the role of group B vitamins and amino acid cross-feeding among marine prokaryotic communities, the results revealed that genome donors occupied significantly more central positions in the ecological network, highlighting their importance among natural communities as well as their potential influence on community assembly. Approaches bypassing the need for cultivating species and enabling such predictions at the community level are promising and, when applied on environmental genomes from phytoplankton species (e.g. SAGs or MAGs), will better improve our mechanistic understanding of metabolic interdependencies and cross-feeding in phytoplankton-associated communities at a larger scale. However, many challenges remain, such as how to link network and ecosystem properties, because experimental evidence is rare and not always in agreement with theoretical expectations [102]. Addressing such issues will allow us to better identify biotic interactions and improve the success rate of validation experiments in interaction discovery [82].

## 4. Future directions

When taken separately, the above methods can provide partial answers to the question ‘who makes what, for whom and when’. While empirical methods represent the gold standard for demonstrating metabolic interactions, these remain difficult to perform in an automated manner, limiting their scale; whereas more *in silico* approaches can process more information and scale up to the community level but are constrained by the availability and quality of reference databases and standardized meta-data, and are easily prone to spurious interpretations. Going deeper into the characterization and understanding of phytoplankton metabolic interactions at the community level would therefore require application of a combination of methods to go back and forth between *in silico* predictions and formal observations. In this last part, we present several studies that have shown promising results that we envision will inspire future studies.

### (a) New techniques to explore both subcellular and community levels

In a pioneering investigation of photosymbioses, Uwizeye *et al*. compared free-living and symbiotic stages of the haptophyte *Phaeocystis* by combining three-dimensional subcellular imaging, photophysiology, carbon flux imaging and transcriptomics [103]. The results showed a disappearance of both external scales and flagella together with dramatic biovolume and nucleus increases in symbiotic *Phaeocystis* cells. Combined with transcriptomes highlighting that genes involved in cell cycle and DNA replication were downregulated, and that genes responsive to nutrient depletion were not upregulated, these analyses pointed towards inhibited cell division in symbiotic *Phaeocystis* in a nutrient-independent process. In contrast, the dramatic expansion of the number and size of symbiont plastids, with the observation of maintained plastid division decoupled from cell division, led, in turn, to enhanced symbiont photosynthetic performance and carbon fixation. Using nanoSIMS analyses, the authors furthermore demonstrated that photosynthetically fixed carbon was transferred to the host. Although the processes that were revealed appeared to be nutrient-independent, this combination of single-cell approaches with high-throughput methodologies can potentially investigate large-scale associations and metabolic interactions especially between hosts and symbionts and/or parasites. Constraints remain regarding the widespread applicability of such a powerful approach to routine fieldwork, notably regarding its associated cost and the reality that sampling is labour intensive and many species are fragile, which makes it difficult to perform high-throughput imaging. We can expect that the application of three-dimensional imaging to larger communities following improvements in technology and methods, e.g. by combining it with automated high-throughput cell sorting on natural samples, will allow the creation of databases of plankton associations from a wide range of ecosystems.

### (b) Combining ‘omics to explore target metabolites and functions within communities

Recent studies have proven effective at disentangling the metabolic currencies exchanged between phytoplankton and their associated microbes using a combination of finely controlled culturing techniques, ‘omics analyses and metabolite measurements. Among the most successful and innovative example studying plankton interactions is the work of Durham *et al.*, in which a combination of field metatranscriptomics, metabolomics and bioinformatics, together with growth assays in the laboratory, were applied to determine the nature of organic sulphur compounds—known as sulfonates—exchanged between eukaryotic phytoplankton and heterotrophic bacteria [104]. The authors first performed a screen of the particulate pools of sulphur-based metabolites in 36 lineages of both eukaryotic and prokaryotic plankton using metabolomics (both targeted and untargeted) that they combined with bioinformatic searches in public genomic and transcriptomic databases of eukaryotic phytoplankton sequences to detect homologues to verified prokaryotic sequences involved in sulfonate metabolism, allowing identification of candidates involved in the target functions across lineages. In parallel, field experiment metatranscriptomes both from eukaryotic phytoplankton and prokaryotes were interrogated to explore the pervasiveness of the candidate genes over a diel cycle, as sulphate assimilation is known to be tightly coupled with circadian rhythms. A combination of these measurements together with the quantification of particulate sulfonates allowed them to distinguish lineage-specific patterns of transcript abundances. Combined with laboratory validation, the results provided evidence for proposed biosynthetic pathways, notably regarding the production of 2,3-dihydroxypropane-1-sulfonate (DHPS), a sulfonate known to support bacterial growth in the oceans, by haptophytes and stramenopiles. Comparisons with prokaryotic catabolic routes using metatranscriptomes highlighted an ecological partitioning of sulfonate degradation: while SAR11 transcripts suggested degradation of DHPS into pyruvate, both SAR116 and Rhodobacterales displayed an additional gene repertoire for degradation of sulfoacetaldehyde and cysteate. Therefore, combining experimental evidence in controlled laboratory conditions with field experiments and community ‘omics analyses allows exploration of the role of specific compounds in structuring microbial plankton communities.

**Figure 2.**
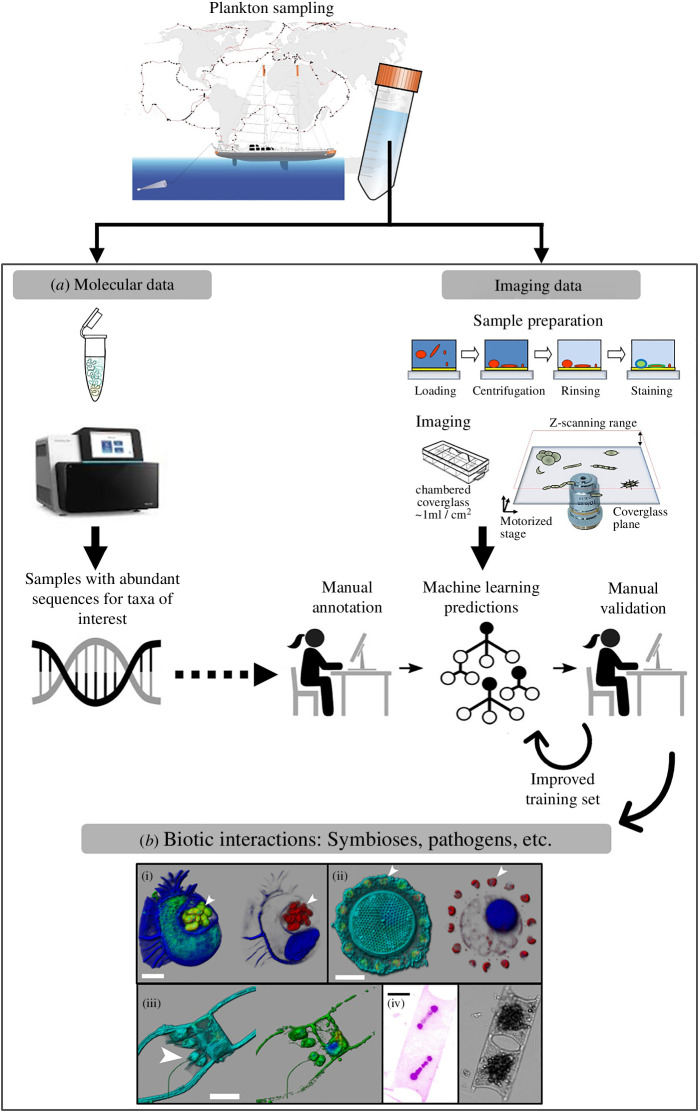
Workflow for the integration of molecular and imaging data to study potential biotic interactions within plankton communities. (*a*) The strategy starts with the mining of molecular data (e.g. metagenomes, metabarcoding) to select a few samples where sequences of interest are abundant. This should then be completed manually by searching through the corresponding databases for the images from those samples—here the quantitative microscopy images (https://ecotaxa.obs-vlfr.fr/) that were generated from environmental high content fluorescence microscopy (e-HCFM) [105]. After obtaining a few manually annotated images, predictions are run for the whole dataset (all samples) using machine learning and curated by visual inspection. These results are then considered as a new training set for running new predictions. Both molecular and imaging results are then combined to verify the predictions and to quantify potential biases (e.g. the variations in gene or genome copies per cell) and to determine biological properties such as the number of endosymbionts/parasites per host cell. (*b*) Examples here include symbioses of the dinoflagellate *Citharistes* sp. (i) that harbours cyanobacteria (arrow head); the diatom *Thalassiosira* sp. (ii) with a belt chain of coccolithophores (*Reticulofenestra sessilis*, arrow head); the diatom *Chaetoceros simplex* (iii) with unidentified epiphytic nanoflagellates (arrow head); and the diatom *Hemiaulus* sp. (iv) and the cyanobacteria *Richelia* sp. as seen through different channels (green, cellular membranes (DiOC6(3)) and core cell bodies; blue, DNA (Hoechst) and thecate dinoflagellate cell wall; red, chlorophyll autofluorescence; cyan, for eukaryotic cell surface (PLL-A546)). Scale bar is 10 µm. Figure adapted and modified from [105,106] based on *Tara* Oceans samples.

It remains challenging to gain insights into global metabolic interactions of phytoplankton within their natural assemblages using ‘omics patterns without neglecting physiological and/or morphological aspects. Recent studies have tackled this problem by combining *in silico* data mining of both molecular and imaging datasets from the *Tara* Oceans databases (figure 2) [105,106]. By bringing together experts on metagenomics and imaging, one study succeeded to show, among other results, the concordant distributions of symbiotic diazotrophs within diatom cells, supported by many images sampled across the world ocean, and of the *nifH* gene, a marker gene of biological N_2_ fixation [107]. Using over 2 000 000 images and PCR-free molecular markers from 1300 metagenomes, several new oceanic regions with high numbers of diazotrophs were discovered, highlighting new sources and sinks of diazotroph-fuelled production, as well as new symbioses that were later confirmed by the isolation of both organisms [108]. The originality of such an approach resides in its multidisciplinarity: while the sequence reads are proxies of the contribution of specific organisms and their distribution within plankton communities, the application of an image classification model on images from an Underwater Vision Profiler—a device developed to quantify the vertical distribution of large particles and plankton (>100 µm in size) at the same time in a known volume of water and that can be mounted on a standard rosette frame—and confocal microscopy allowed the linking of genomics to the images and *vice versa*, which revealed remarkably congruent patterns (figure 2). Most notably, these processes provided the first estimation of diazotroph biovolumes and allowed the integration of their ecological complexity (i.e. symbiotic, colony-forming, particle-associated and unicellular). In turn, the functional diversity of the nitrogen-fixing community in these samples was described in a subsequent work based on the reconstruction of MAGs, which also identified species with metabolic potential for the synthesis of group B vitamin [88]. However, it is still necessary to measure actual rates of nitrogen fixation in the field to get a full picture of the process. This gene-imaging workflow is directly applicable to other microbial populations. As an example, marine picocyanobacteria were typically considered to live an exclusively single-celled planktonic lifestyle, but the recent combination of microscopy and the photosynthetic marker gene *psbO* showed that colonial and symbiotic forms are widespread in the ocean [109]. Metabolic interactions are still to be explored in these picocyanobacterial lifestyles, but chitin degradation pathways seems to play a role [110]. However, many limitations remain, notably since collections represent snapshots of the community at a given time, which can make extrapolations difficult. One challenge will be to adapt and standardize the analytical pipelines in which the samples can be easily processed by different methodologies to more accurately represent species diversity, both qualitatively and quantitatively. Furthermore, as the resulting molecular sequences are taxonomically classified by comparisons with reference sequence databases, the relevance of the results relies heavily on the database quality [106]. As for sequences, the inherent importance of the training sets for machine learning methodologies must be noted because database limitations affect the annotation of high-throughput images. To provide efficient classification and replicability, large, diverse, balanced and high-quality databases are required, with realistic proportions of taxa. Intercomparisons of future projects with past and ongoing research programmes aiming at expanding plankton databases in both marine [111] and freshwater [112] environments will be pivotal for methodological improvement.

## 5. Conclusion

The exploration of phytoplankton metabolic interactions at the community level requires a trade-off between complexity level and tractability. In this regard, moving back and forth between culture-based and *in silico* approaches appears pivotal when trying to integrate results from local to global scales, and *vice versa*. Although purely *in silico* predictive approaches may at first seem far away from what is observed in natural populations, deriving information from networks and models is an important way to generate new hypotheses that can be tested in the laboratory. Cross-disciplinary research is thus required to move forward, and we consider that global projects gathering the scientific community around common research questions and objectives, such as the *Tara* Oceans project, can represent a useful model.

Because a better understanding of the ecology and biodiversity of phytoplankton and their plankton partners appears an essential task of the UN Decade for Ocean Science [113,114], future global sequencing and monitoring programmes must involve participation from researchers in transitional and developing economies that are likely to harbour highly diverse plankton communities that will be disproportionately affected by anthropogenic climate change. In parallel, new initiatives to promote citizen-based research at a planetary scale are being developed. Among them, the Plankton Planet programme derived from *Tara* Oceans is envisioned as a collaborative effort between scientists and sailors to develop cost-effective instruments, such as the PlanktoScope [115], and deploy them in sea-faring communities to conduct a homogeneous global plankton survey [116]. Such initiatives will enable the generation of standardized biological data, both qualitative and quantitative, allowing a systematic assessment of plankton life across defined spatio-temporal scales. These approaches will generate new data to help predict the emerging functions and evolution of ecosystems.

Marine ecosystems are in constant balance between carbon production and transfer to higher trophic levels, remineralization and export to deeper layers and they are thus dependent on a wide range of biotic interactions [77,117]. Global efforts combining metabarcoding and gene correlation networks notably revealed the significant correlation of dinoflagellates and predicted parasites of plankton correlate significantly with carbon export in the oligotrophic open ocean [118]. Biological functions dedicated to photosynthesis and growth were highlighted as the most associated with carbon export, while many others remain to be characterized. On the other hand, recent studies applying cell sorting methodologies that circumvent disruption of cell associations during sampling revealed a new range of interactions between choanoflagellates—widespread protists known to consume bacteria—and uncultured endosymbiotic bacteria showing significant genome streamlining for functions as important as glycolysis and cell wall synthesis, further blurring the lines between the concepts of predator–prey interactions and symbioses and their implications for global carbon and energy transfer [119]. Gaining knowledge on the metabolic interactions between phytoplankton and their partners is therefore mandatory to provide answers about their broader life strategies and contribution to fluxes of matter and energy, which could, in turn, refine our knowledge of how these organisms contribute to trophic networks and global biogeochemical cycles, fuelling larger ecological models [117]. One way to effectively integrate the product of metabolic interactions into these fluxes is through the estimation of biomass and biovolume. While biomass refers to the total quantity of organisms in a given volume or area, the biovolume represents the volume of their cells and is a surrogate for estimating their biomass—a measure particularly important to explore the fluxes of matter and energy. Using metabarcoding as a proxy of biovolume—an approach with significant bias [120]—remains very challenging for phytoplankton [121] and further development is required to validate new marker genes, although results are improved when combined with imaging [109].

As environmental conditions strongly influence plankton distribution, climate change is expected to have major consequences for community composition and biogeography [122,123], which will, in turn, impact their ecology. Global diversity and environmental niche models predict a potential diversity decline [124,125], while a 4% reduction of carbon export fluxes owing to phytoplankton and copepod community changes is expected by the end of the century [126]. By better integrating environmental constraints of predicted climate change scenarios (e.g. in culture conditions in the lab, mesocosm experiments or metabolic models), methods exploring phytoplankton metabolic interactions will help infer their pertaining impact to global marine trophic networks.

## Data Availability

This article has no additional data.
